# Supplementary material for: Effect of Oral Nutritional Supplements with Sucromalt and Isomaltulose versus Standard Formula on Glycaemic Index, Entero-Insular Axis Peptides and Subjective Appetite in Patients with Type 2 Diabetes: A Randomised Cross-Over Study
Source: Nutrients. 2019 Jun 28;11(7):1477. doi: 10.3390/nu11071477 (PMC6683048; doi:10.3390/nu11071477)
Supplement: Supplementary file 1 [file nutrients-11-01477-s001.pdf]

Table S1. Correlations coefficients between biochemical variables and subjective measurements of appetite in 0, 30, 90 and 120 min at standard oral nutritional supplement not specific for diabetic patients (ET).

	Time (min)	Hunger (mm)		Fullness (mm)		Desire to eat (mm)		Prospective food consumption (mm)		Subjective average appetite	
		r	*p*	*r*	*p*	*r*	*p*	*r*	*p*	*r*	*p*
**Glycaemia (mg/dL)**	T0	0.138	0.703	0.047	0.897	0.428	0.217	0.076	0.834	0.521	0.122
	T30	-0.264	0.461	-0.010	0.979	-0.233	0.517	-0.568	0.086	0.570	0.085
	T90	-0.362	0.304	-0.285	0.426	0.069	0.850	-0.221	0.540	0.040	0.912
	T120	-0.456	0.185	-0.736	0.015	0.005	0.990	0.478	0.162	0.575	0.082
**Insulin (mU/L)**	T0	0.276	0.440	0.162	0.655	0.385	0.272	-0.468	0.172	-0.072	0.842
	T30	-0.745	0.012	0.572	0.084	-0.204	0.571	-0.036	0.922	-0.849	0.002
	T90	-0.108	0.767	0.143	0.693	0.222	0.538	-0.031	0.932	-0.086	0.812
	T120	-0.078	0.831	0.270	0.451	0.541	0.106	-0.490	0.151	-0.279	0.436
**GLP-1 (pmol/L)**	T0	-0.210	0.560	-0.359	0.308	-0.138	0.703	0.048	0.896	-0.068	0.852
	T30	-0.133	0.714	-0.220	0.542	0.039	0.914	0.124	0.732	0.090	0.805
	T90	0.395	0.259	-0.513	0.130	0.312	0.379	0.015	0.968	0.552	0.098
	T120	-0.194	0.592	-0.417	0.231	-0.513	0.130	-0.094	0.797	-0.032	0.931
**GIP (pg/ml)**	T0	0.200	0.579	0.312	0.381	-0.553	0.097	0.270	0.451	-0.038	0.970
	T30	-0.519	0.124	0.124	0.733	-0.293	0.412	0.036	0.922	-0.481	0.159
	T90	-0.323	0.362	0.381	0.278	0.206	0.567	0.360	0.306	-0.135	0.711
	T120	-0.196	0.587	-0.089	0.808	-0.216	0.548	-0.271	0.450	-0.285	0.424

GIP: glucose-dependent insulinotropic polypeptide; GLP-1: glucagon-like peptide 1. Values presented correspond to *r coefficients* and *p-value* for all subject correlations between subjective perceptions of appetite and concentrations hormones according to the treatment group. *p-value* was significant when less than 0.05.

Table S2. Correlations coefficients between biochemical variables and subjective measurements of appetite in 0, 30, 90 and 120 min at isomaltulose and resistant starch supplement (DI).

	Time (min)	Hunger (mm)		Fullness (mm)		Desire to eat (mm)		Prospective food consumption (mm)		Subjective average appetite	
		r	*p*	*r*	*p*	*r*	*p*	*r*	*p*	*r*	*p*
**Glycaemia (mg/dL)**	T0	-0.727	0.017	-0.166	0.647	-0.656	0.039	-0.309	0.384	-0.826	0.003
	T30	0.164	0.650	-0.202	0.577	0.125	0.731	0.775	0.008	0.526	0.118
	T90	0.258	0.471	0.149	0.681	-0.235	0.514	0.538	0.108	0.174	0.630
	T120	0.370	0.293	0.236	0.512	-0.259	0.469	-0.148	0.684	-0.185	0.610
**Insulin (mU/L)**	T0	-0.079	0.828	-0.686	0.028	-0.036	0.922	0.048	0.895	0.307	0.389
	T30	0.382	0.277	-0.188	0.602	-0.083	0.819	0.126	0.728	-0.097	0.790
	T90	0.173	0.633	-0.179	0.620	-0.359	0.309	0.090	0.804	0.041	0.911
	T120	-0.053	0.884	-0.033	0.928	-0.629	0.051	-0.224	0.534	0.445	0.197
**GLP-1 (pmol/L)**	T0	-0.650	0.042	0.131	0.719	-0.087	0.811	0.386	0.271	-0.262	0.465
	T30	-0.560	0.092	0.375	0.286	0.269	0.453	0.082	0.821	-0.212	0.556
	T90	0.395	0.259	-0.089	0.808	0.268	0.455	0.370	0.293	0.529	0.116
	T120	0.280	0.434	-0.084	0.818	-0.667	0.035	0.073	0.841	-0.106	0.770
**GIP (pg/ml)**	T0	0.791	0.006	-0.094	0.797	-0.076	0.834	-0.073	0.842	0.403	0.249
	T30	-0.385	0.272	0.003	0.993	-0.610	0.061	-0.416	0.231	-0.688	0.028
	T90	0.252	0.483	-0.232	0.519	-0.130	0.720	0.093	0.798	-0.318	0.370
	T120	0.432	0.213	0.363	0.302	-0.200	0.579	-0.261	0.466	-0.270	0.154

GIP: glucose-dependent insulinotropic polypeptide; GLP-1: glucagon-like peptide 1. Values presented correspond to *r coefficients* and *p-value* for all subject correlations between subjective perceptions of appetite and concentrations hormones according to the treatment group. *p-value* was significant when less than 0.05.

Table S3. Correlations coefficients between biochemical variables and subjective measurements of appetite in 0, 30, 90 and 120 min at resistant maltodextrin and sucromalt supplement (GS).

	Time (min)	Hunger (mm)		Fullness (mm)		Desire to eat (mm)		Prospective food consumption (mm)		Subjective average appetite*	
		r	*p*	*r*	*p*	*r*	*p*	*r*	*p*	*r*	*p*
**Glycaemia (mg/dL)**	T0	0.124	0.733	0.057	0.876	-0.267	0.457	0.164	0.650	-0.017	0.963
	T30	0.737	0.011	-0.138	0.703	0.378	0.282	0.294	0.410	0.711	0.021
	T90	-0.076	0.835	0.698	0.025	0.379	0.281	-0.224	0.534	-0.332	0.348
	T120	0.310	0.383	-0.148	0.683	-0.069	0.850	0.450	0.192	0.410	0.239
**Insulin (mU/L)**	T0	-0.364	0.301	-0.665	0.036	-0.573	0.710	0.055	0.881	0.065	0.859
	T30	0.515	0.127	-0.373	0.289	0.441	0.202	-0.289	0.418	0.562	0.091
	T90	-0.289	0.419	-0.004	0.992	-0.268	0.454	0.206	0.567	-0.174	0.631
	T120	0.451	0.191	0.061	0.866	0.523	0.121	-0.251	0.484	0.258	0.471
**GLP-1 (pmol/L)**	T0	-0.162	0.655	0.253	0.481	-0.195	0.590	0.214	0.553	-0.274	0.444
	T30	0.454	0.187	0.167	0.644	0.585	0.076	-0.722	0.018	0.269	0.452
	T90	-0.194	0.592	-0.454	0.188	-0.364	0.301	0.442	0.201	0.174	0.630
	T120	-0.117	0.748	0.250	0.486	0.100	0.784	-0.538	0.108	-0.393	0.261
**GIP (pg/ml)**	T0	0.133	0.714	-0.735	0.015	-0.356	0.313	0.023	0.949	0.467	0.170
	T30	-0.067	0.854	0.446	0.197	-0.165	0.648	-0.081	0.824	-0.407	0.242
	T90	0.825	0.003	0.151	0.676	0.019	0.958	-0.252	0.482	0.244	0.496
	T120	0.266	0.458	-0.113	0.407	0.449	0.193	-0.116	0.750	0.298	0.403

GIP: glucose-dependent insulinotropic polypeptide; GLP-1: glucagon-like peptide 1. Values presented correspond to *r coefficients* and *p-value* for all subject correlations between subjective perceptions of appetite and concentrations hormones according to the treatment group. *p-value* was significant when less than 0.05.

Table S4. Correlations coefficients between glycemic index and glycaemia load with AUC values of hormones and subjective measurements of appetite.

	GI		GL	
**ET intervention**	***r***	***p***	***r***	***p***
Insulin	-0.144	0.691	-0.144	0.691
GLP-1	-0.022	0.952	-0.022	0.952
GIP	0.112	0.759	0.066	0.856
Hunger	0.777	0.008	0.777	0.008
Fullness	0.283	0.429	0.283	0.429
Desire to eat	0.231	0.520	0.231	0.520
Prospective food consumption	0.018	0.960	0.018	0.960
Subjective average appetite	0.263	0.462	0.263	0.462
**DI intervention**	*r*	*p*	*r*	*p*
Insulin	-0.437	0.207	-0.437	0.207
GLP-1	0.111	0.761	0.111	0.761
GIP	-0.867	0.001	-0.867	0.001
Hunger	-0.071	0.845	-0.071	0.845
Fullness	-0.057	0.875	-0.057	0.875
Desire to eat	0.519	0.124	0.519	0.124
Prospective food consumption	-0.370	0.292	-0.370	0.292
Subjective average appetite	0.353	0.316	0.353	0.316
**GS intervention**	*r*	*p*	*r*	*p*
Insulin	-0.063	0.863	-0.063	0.863
GLP-1	0.087	0.811	0.087	0.811
GIP	-0.892	0.001	-0.892	0.001
Hunger	-0.458	0.183	-0.458	0.183
Fullness	-0.698	0.025	-0.698	0.025
Desire to eat	-0.623	0.054	-0.623	0.054
Prospective food consumption	0.242	0.501	0.242	0.501
Subjective average appetite	-0.117	0.747	-0.117	0.747

Treatment groups were defined as a standard nutritional supplement not specific for people with diabetes (ET); resistant maltodextrin and sucromalt supplement (GS); isomaltulose and resistant starch supplement (DI). GIP: glucose-dependent insulinotropic polypeptide; GLP-1: glucagon-like peptide 1. Values presented correspond to *r coefficients* and *p-value* for all subject correlations between levels ($AUC_{0\text{-}180min}$) of subjective perceptions of appetite and concentrations hormones according to the treatment group. *p-value* was significant when less than 0.05.
